# Acute Peripheral but Not Central Administration of Olanzapine Induces Hyperglycemia Associated with Hepatic and Extra-Hepatic Insulin Resistance

**DOI:** 10.1371/journal.pone.0043244

**Published:** 2012-08-14

**Authors:** Elodie M. Girault, Anneke Alkemade, Ewout Foppen, Mariëtte T. Ackermans, Eric Fliers, Andries Kalsbeek

**Affiliations:** 1 Hypothalamic Integration Mechanisms, Netherlands Institute for Neuroscience, an Institute of the Royal Netherlands Academy of Arts and Science, Amsterdam, The Netherlands; 2 Department of Endocrinology and Metabolism, Academic Medical Center (AMC), University of Amsterdam, Amsterdam, The Netherlands; 3 Laboratory of Endocrinology, Department of Clinical Chemistry, Academic Medical Center (AMC), University of Amsterdam, Amsterdam, The Netherlands; Max-Delbrück Center for Molecular Medicine (MDC), Germany

## Abstract

Atypical antipsychotic drugs such as Olanzapine induce weight gain and metabolic changes associated with the development of type 2 diabetes. The mechanisms underlying the metabolic side-effects of these centrally acting drugs are still unknown to a large extent. We compared the effects of peripheral (intragastric; 3 mg/kg/h) versus central (intracerebroventricular; 30 µg/kg/h) administration of Olanzapine on glucose metabolism using the stable isotope dilution technique (Experiment 1) in combination with low and high hyperinsulinemic-euglycemic clamps (Experiments 2 and 3), in order to evaluate hepatic and extra-hepatic insulin sensitivity, in adult male Wistar rats. Blood glucose, plasma corticosterone and insulin levels were measured alongside endogenous glucose production and glucose disappearance. Livers were harvested to determine glycogen content. Under basal conditions peripheral administration of Olanzapine induced pronounced hyperglycemia without a significant increase in hepatic glucose production (Experiment 1). The clamp experiments revealed a clear insulin resistance both at hepatic (Experiment 2) and extra-hepatic levels (Experiment 3). The induction of insulin resistance in Experiments 2 and 3 was supported by decreased hepatic glycogen stores in Olanzapine-treated rats. Central administration of Olanzapine, however, did not result in any significant changes in blood glucose, plasma insulin or corticosterone concentrations nor in glucose production. In conclusion, acute intragastric administration of Olanzapine leads to hyperglycemia and insulin resistance in male rats. The metabolic side-effects of Olanzapine appear to be mediated primarily via a peripheral mechanism, and not to have a central origin.

## Introduction

Atypical antipsychotic drugs (AAPDs) are increasingly replacing the use of typical antipsychotics due to their decreased risk for extrapyramidal side-effects [1] and their higher efficacy in the treatment of negative symptoms of schizophrenia [2]–[4]. However, some AAPDs are associated with unfavorable metabolic side-effects such as weight gain and insulin resistance [3], [5]. Epidemiologic studies showed that Olanzapine is one of the AAPDs that causes the most pronounced weight gain [6]. Also clinically, Olanzapine represents one of the atypical antipsychotics with the greatest risk of inducing weight gain and/or metabolic disturbances [7]. These metabolic side-effects, especially weight gain, decrease patients compliance [8] even though Olanzapine is a very effective drug in terms of symptom reduction [4], [9]. Moreover, weight gain and insulin resistance are risk factors for type 2 diabetes and cardiovascular diseases [10].

The mechanisms underlying Olanzapine-induced metabolic disturbances are still unclear. Olanzapine is known to bind to a great number of receptors, such as the histamine H1 receptor [11], [12], the serotonin 5-HT2c receptor [13]–[15], the adrenergic α2 and β3 receptors, the acetylcholine m3 receptor (high affinity) and the dopamine 2 (D2) receptor (low affinity) [16], [17]. In addition, it is not obvious whether the metabolic side-effects are mediated by central or peripheral effects of the drug. The principal mechanism of action of AAPDs is clearly based on their actions in the central nervous system (CNS), but the receptors they bind to are also widely expressed in peripheral tissues such as the liver [18]–[22].

**Figure 1:**
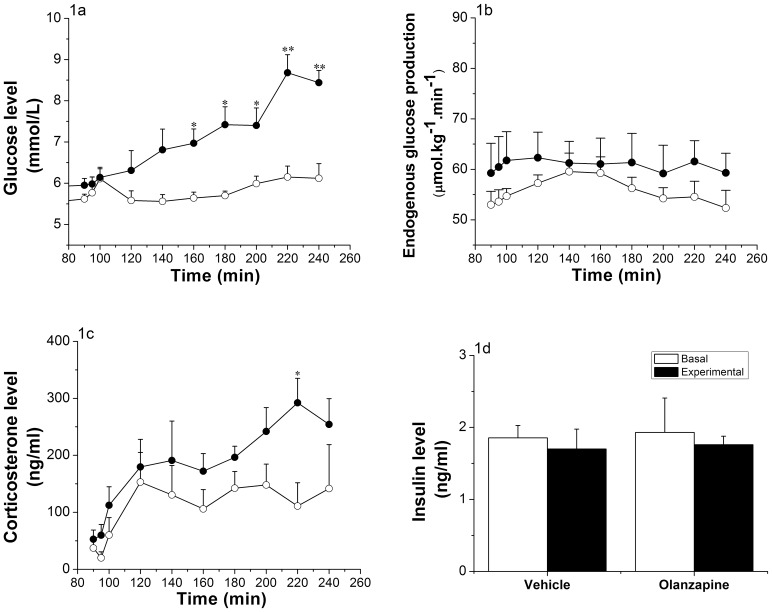
Effects of intragastric infusion of Olanzapine. (Vehicle group n = 5, Olanzapine group n = 6). 1a: Glucose evolution before (t = 90 to t = 100) and during (t = 120 to t = 240) the intragastric infusion of Olanzapine infusion (3 mg/kg/h). Glycemia significantly increased after 60 min of Olanzapine infusion (*p<0.05, **p<0.001; ANOVA repeated measures; *Time,* p<0.001; Time * Group, p<0.001; *Group,* p = 0.001). 1b: Endogenous glucose production before (t = 90 to t = 100) and during (t = 120 to t = 240) intragastric Olanzapine infusion. No significant changes were observed (ANOVA repeated measures; *Time,* p = 0.426; Time * Group, p = 0.937; *Group,* p = 0.356). 1c: Corticosterone levels before (t = 90 to t = 100) and during (t = 120 to t = 240) intragastric Olanzapine infusion. Corticosterone levels are significantly higher in the Olanzapine group only at t = 220 (*p<0.05; ANOVA repeated measures; *Time,* p<0.001; Time * Group, p = 0.58; *Group,* p = 0.039). 1d: Plasma insulin levels before (mean of time points t = 90 and t = 100) and at the end (mean of time points t = 180 and t = 220) of the intragastric infusion of Olanzapine. No significant differences were detected (ANOVA repeated measures; *Time,* p = 0.601; Time * Group, p = 0.981; *Group,* p = 0.834). Vehicle-treated animals  =  white dots; Olanzapine-treated animals  =  black dots.

In rat models, it has been shown that an acute subcutaneous administration of Olanzapine induces insulin resistance by increasing hepatic glucose production and decreasing glucose uptake [23]. These findings indicate that the Olanzapine-induced metabolic changes can occur rapidly, and even before the weight gain occurs, indicating that these effects are not secondary to the weight gain. Two studies investigated possible central effects of Olanzapine using acute intracerebroventricular (ICV) infusions of the drug, however, whilst one study reported Olanzapine to induce metabolic changes [24] the other study found no metabolic changes after the central administration [25]. Neither of these two studies published plasma levels of Olanzapine post-infusion, therefore a possible peripheral effect due to leakage cannot be excluded.

In order to elucidate the metabolic side-effects of Olanzapine and the mechanism thereof, we compared the acute effects of a peripheral (intragastric, IG) versus a central (ICV) administration of Olanzapine on glucose production and insulin sensitivity using the stable glucose isotope dilution technique in combination with hyperinsulinemic-euglycemic clamps.

## Materials and Methods

### Ethic Statement

All experiments were approved by the animal care committee of the Royal Netherlands Academy of Arts and Science.

### Animals

Male Wistar rats (Harlan Nederland, Horst, The Netherlands) weighing 300–350 g were individually housed (cages 40×25×25 cm) and maintained on a 12 h/12 h light/dark cycle (lights on at 7:00 am) at 21±1°C and 60±5% relative humidity. Food (standard rodent chow, Teklad) and water were available ad libitum. Experiments 1, 2 and 3 have been performed on separate sets of animals.

**Figure 2:**
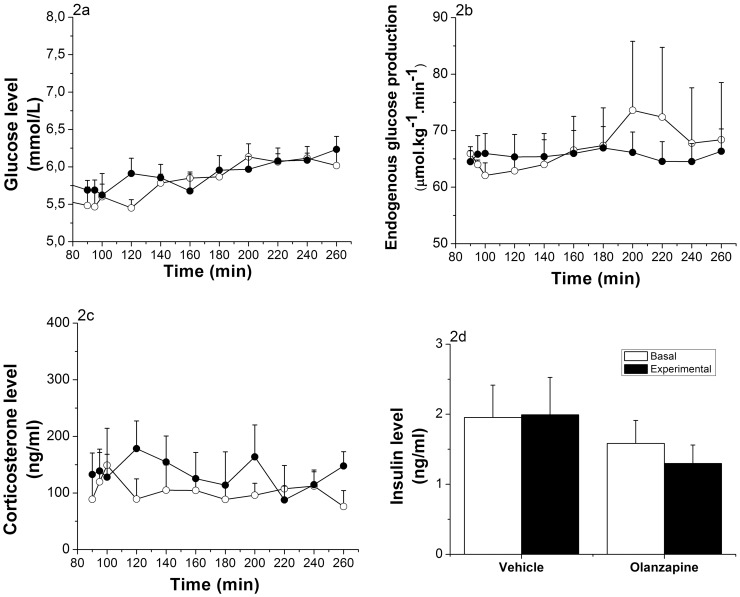
Effects of ICV infusion of Olanzapine. (Vehicle group n = 6, Olanzapine group n = 9). 2a: Glucose evolution before (t = 90 to t = 100) and during (t = 120 to t = 260) ICV Olanzapine infusion (30 µg/kg/h). No significant differences between the 2 groups were detected (ANOVA repeated measures; *Time,* p<0.001; Time * Group, p = 0.59; *Group,* p = 0.635). 2b: Endogenous glucose production before (t = 90 to t = 100) and during (t = 120 to t = 260) ICV Olanzapine infusion. No significant changes were detected (ANOVA repeated measures; *Time,* p = 0.731; Time * Group, p = 0.709; *Group,* p = 0.84). 2c: Corticosterone levels before (t = 90 to t = 100) and during (t = 120 to t = 260) ICV Olanzapine infusion. No significant changes were detected (ANOVA repeated measures; *Time,* p = 0.971; Time * Group, p = 0.631; *Group,* p = 0.546). 2d: Plasma insulin levels before (mean of time points t = 90 and t = 100) and at the end (mean of time points t = 220 and t = 260) of the ICV infusion of Olanzapine. No significant changes were detected (ANOVA repeated measures; *Time,* p = 0.722; Time * Group, p = 0.638; *Group,* p = 0.274). Vehicle-treated animals  =  white dots; Olanzapine-treated animals  =  black dots.

### Drugs

The dose of Olanzapine chosen in the present studies was selected to parallel the clinical setting based on 70% dopamine D2 receptor occupancy, which represents a threshold in humans associated with optimal clinical response [16]. The route of administration was chosen such that a continuous infusion of freshly made solution was possible in freely moving, undisturbed animals. Using a surgically implanted intragastric catheter, animals were treated with a primed 36 mg/kg/h infusion during 5 minutes followed by a continuous 3 mg/kg/h infusion for 160 minutes (i.e., in total 3.66 mg/rat) of Olanzapine (ChemPacific Corporation, Maryland) dissolved in acidified MilliQ water (pH = 6). A second set of animals were treated with 360 µg/kg/h during 5 minutes and 30 µg/kg/h for 160 minutes (i.e., in total 36.6 µg per rat) administered via an ICV cannula, representing 1% of the peripheral dose. Solubility of the drug when applied in the ventricular compartment was tested separately and appeared to be maintained when added to artificial cerebrospinal fluid at physiological pH. Olanzapine solution for intragastric infusion was prepared in milliQ water acidified with HCl (1 M) and then brought back to pH 6 using NaOH (1 M).

**Figure 3:**
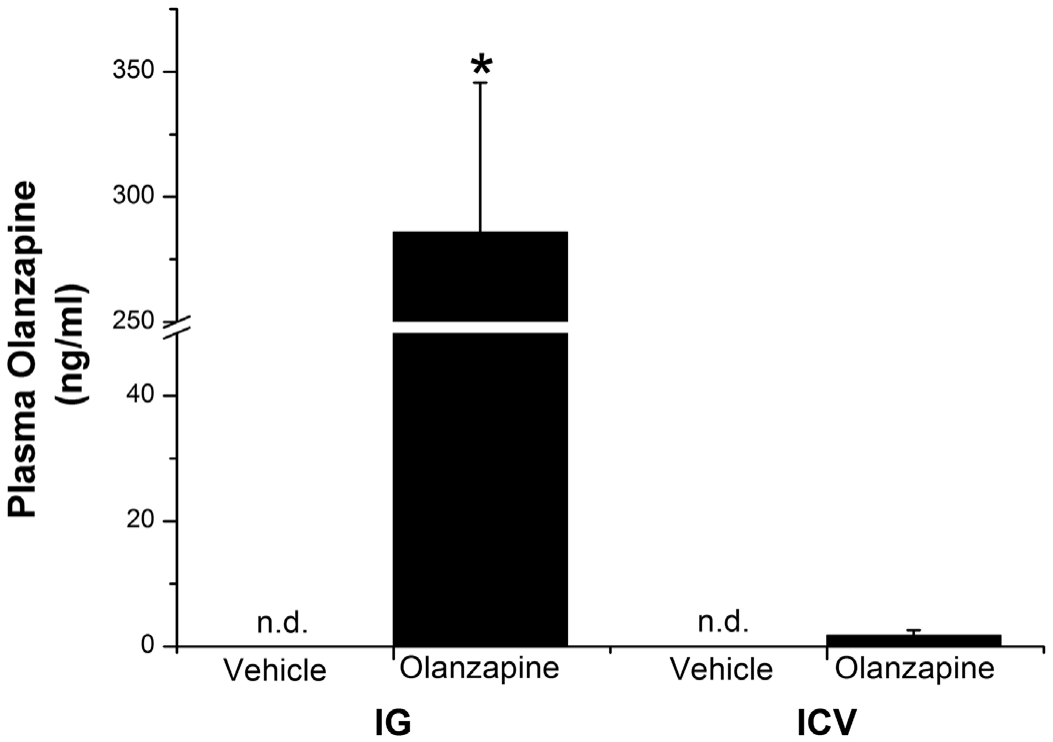
Plasma Olanzapine levels after intragastric (IG) and intracerebroventricular (ICV) infusion of Olanzapine. (IG: Vehicle group n = 5, Olanzapine group n = 6 and ICV Vehicle group n = 8 and Olanzapine group n = 12) Plasma Olanzapine levels are significantly higher in IG-Olanzapine-treated than in IG-vehicle-infused animals (One-Way ANOVA, p<0.001), or ICV-Olanzapine animals (2-Way ANOVA, Administration route * Treatment p<0.001). Plasma Olanzapine levels of ICV-Olanzapine animals are not significantly different from the ICV-Vehicle animals (One-Way ANOVA, p = 0.2). Vehicle-treated animals  =  white bars; Olanzapine-treated animals  =  black bars; *p<0.001.

### Surgical Procedures

After 7 days of habituation, animals were anesthetized by an intramuscular injection of 0.9 ml/kg Hypnorm (Janssen, High Wycombe, Buckinghamshire, UK) and a subcutaneous injection of 0.3 ml/kg Dormicum (Roche, Almere, The Netherlands). Silicon catheters were placed into the right jugular vein and the left carotid artery for intravenous infusions and blood sampling. The vascular lines were closed using a mix of polyvinylpyruvidon (PVP; Sigma-Aldrich Corp., St. Louis, MO), heparin and amoxicillin. For the peripheral study, a silicon cannula was placed in the stomach during the same surgery. Intragastric cannulas were placed through a 1 cm incision on the left side of the abdomen. For the central study, ICV probes were placed into the lateral cerebral ventricle using a standard Kopf stereotaxic apparatus (Anteroposterior: −0.8 mm, Lateral: 2.0 mm, Ventral: −3.2 mm, Angle: 0). Catheters and intragastric cannula were fixed on the top of the head of the animal using dental cement. These techniques allowed us to perform all our experiments in freely moving animals. Experiments were performed only after recovery of the pre-surgical bodyweight and with animals in healthy shape, i.e. 7–10 days post-operative recovery.

**Figure 4:**
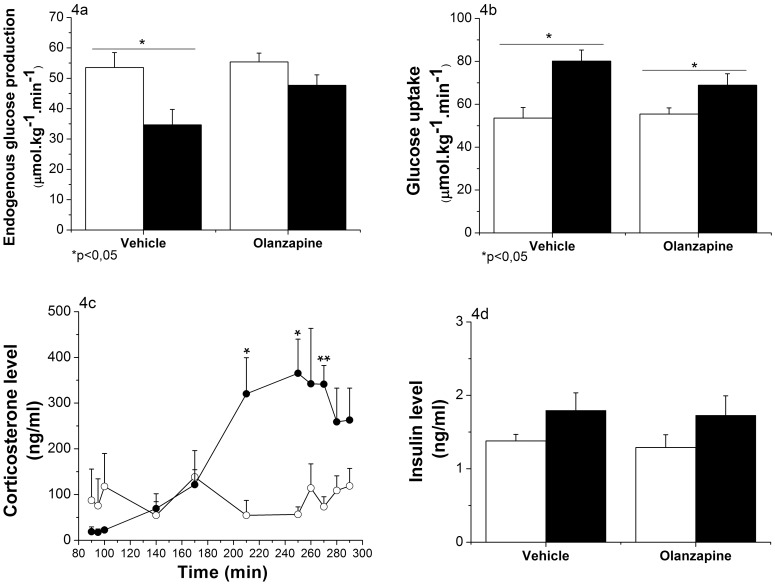
Effects of intragastric infusion of Olanzapine on liver insulin sensitivity (Experiment 2). (Vehicle group n = 8, Olanzapine group n = 7) 4a: Endogenous glucose production at basal (mean of 3 time points: t = 90 to t = 100; white bars) and during the hyperinsulinemic state (mean of 5 time points: t = 250 to t = 290; black bars). EGP significantly decreased during the hyperinsulinemic state for the vehicle group (p = 0.018, One-Way ANOVA) and remained unchanged in the Olanzapine group (p = 0.111, One-Way ANOVA). 4b: Glucose uptake at basal (mean of 3 time points: t = 90 to t = 100; white bars) and during the hyperinsulinemic state (mean of 5 time points: t = 250 to t = 290; black bars). Glucose uptake is significantly increased in both the vehicle (p = 0.002, One-Way ANOVA) and Olanzapine group (p = 0.046, One-Way ANOVA). 4c: Plasma corticosterone levels were significantly elevated by the IG infusion of Olanzapine (ANOVA repeated measures; *Time,* p<0.001; Time * Group, p<0.001; *Group,* p = 0.039). Vehicle-treated animals  =  white dots; Olanzapine-treated animals  =  black dots. 4d: Plasma insulin levels were elevated 1.3-fold during the hyperinsulinemic state (mean of 3 time points; black bars) compared to the basal level (mean of 2 time points; white bars) (ANOVA repeated measures; *Time,* p = 0.062; Time * Group, p = 0.956; *Group,* p = 0.706). *p<0.05,**p<0.001.

### Experimental Procedures

During the experiments, animals were permanently connected to blood-sampling and infusion lines, which were attached to a metal collar and kept out of reach from the rats by means of a counterbalanced arm. This allowed all manipulations to be performed outside the cages without handling the animals. The metal collars were attached the day before the experiment. Before the day of the experiment, food was restricted to 20 g overnight. Two hours before the experiment, rats were handled to connect them to the blood sampling and infusion lines and all remaining food was removed.

**Figure 5:**
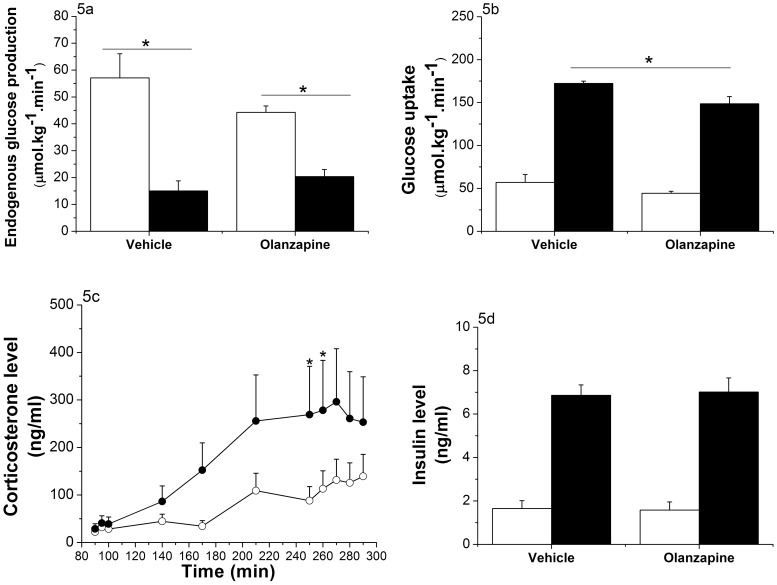
Effects of intragastric infusion of Olanzapine (t = 130 to t = 290; 3 mg/kg/h) on glucose disappearance (Experiment 3). (Vehicle group n = 8, Olanzapine group n = 7) 5a: Endogenous glucose production at basal (mean of 3 time points: t = 90 to t = 100; white bars) and during the hyperinsulinemic state (mean of 5 time points: t = 250 to t = 290; black bars). EGP is significantly decreased in both groups (p<0.001), but the decrease is smaller in the group treated with Olanzapine (ANOVA repeated measures; *Time,* p<0.001; Time * Group, p = 0.018; *Group,* p = 0.604). 5b: Glucose disappearance at basal (mean of 3 time points: t = 90 to t = 100; white bars) and during the hyperinsulinemic state (mean of 5 time points: t = 250 to t = 290; black bars). Glucose disappearance is significantly increased in both groups (ANOVA repeated measures; *Time,* p<0.001; Time * Group, p = 0.475; *Group,* p = 0.005). This increase is significantly smaller for the group treated with Olanzapine (p = 0.014, One-Way ANOVA). 5c: Corticosterone levels are significantly increased by the Olanzapine treatment (ANOVA repeated measures; *Time,* p<0.001; Time * Group, p<0.005; *Group,* p = 0.035). Vehicle-treated animals  =  white dots; Olanzapine-treated animals  =  black dots. 5d: Plasma insulin levels are elevated 4.4-fold in both groups during the hyperinsulinemic state (mean of 3 time points; black bars) compared to the basal level (mean of 2 time points; white bars) (ANOVA repeated measures; *Time,* p<0.001; Time * Group, p = 0.787; *Group,* p = 0.938). *p<0.05.

#### 1. Experiment 1: Basal endogenous glucose production

To assess endogenous glucose production (EGP), [6,6-^2^H_2_]glucose was used as a tracer. Blood samples were taken at t = −5 min for background enrichment (t = 0 was at 11.00 a.m.), at t = 90, t = 95 and t = 100 min to determine enrichment during the equilibrium state and every 20 min from t = 120 till t = 260 to determine enrichment during the experiment.

Vehicle (MilliQ water at pH = 6 to mimic the pH of the Olanzapine solution for the intragastric infusion (1 ml/h) and Ringer for the ICV infusion (5 µl/h)) started together with a continuous [6,6-^2^H_2_]glucose via the jugular vein in both groups at t = 0. After the t = 100 min blood sample (at 12.40 a.m.), vehicle infusion was changed to Olanzapine or vehicle solution (36 mg/kg/h during 5 min and 3 mg/kg/h until the end of the experiment for the intragastric infusion and 360 µg/kg/h during 5 min and 30 µg/kg/h till the end of the experiment for the ICV infusion). At the end of the experiment, animals were sacrificed by a lethal intravenous injection of pentobarbital. Trunk blood was collected for plasma Olanzapine measurements at the end of the experiment. Samples of liver were snap frozen in liquid nitrogen and stored for glycogen measurements.

**Figure 6:**
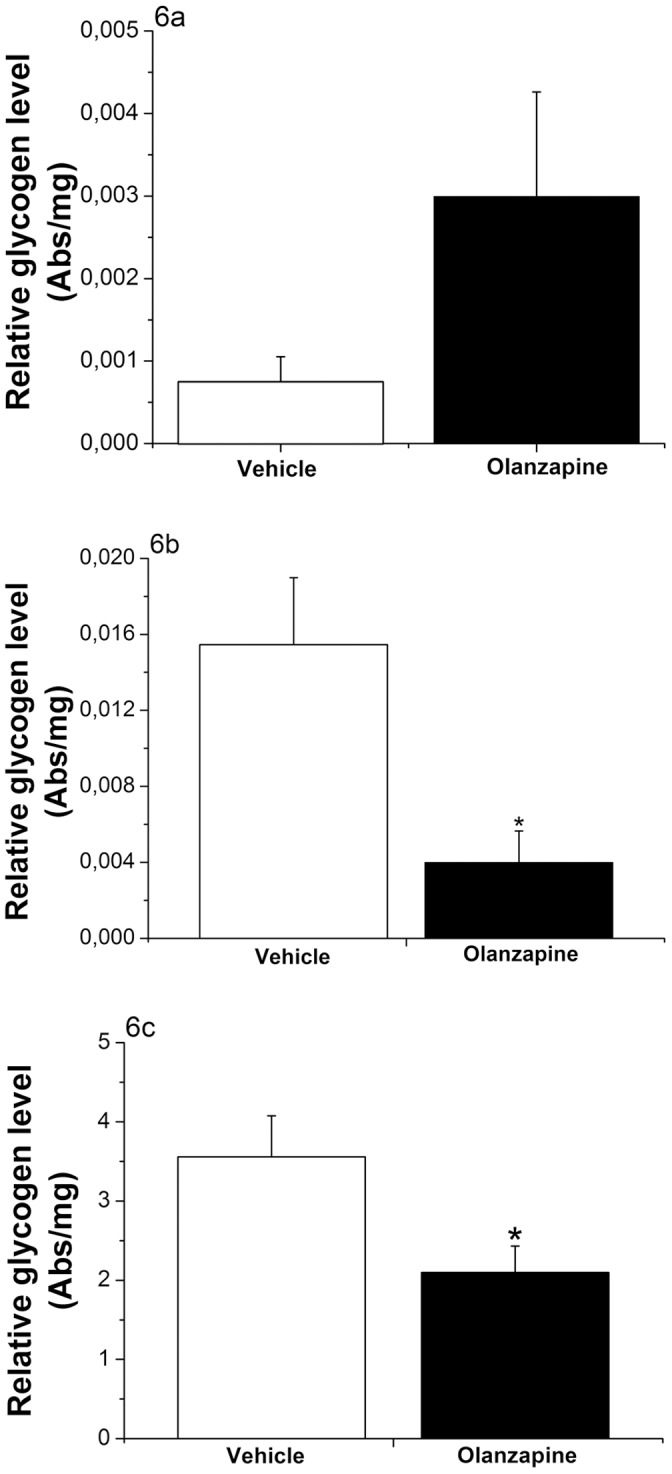
Effects of intragastric infusion of Olanzapine on liver glycogen content in Experiments 1, 2 and 3. 6a: During Experiment-1 hepatic glycogen levels show a non-significant increase in the Olanzapine-treated animals (p = 0.096; T-test). 6b: During the low-level hyperinsulinemic-euglycemic clamp (Experiment-2) hepatic glycogen levels are decreased in the Olanzapine-treated animals (p = 0.007; T-test). 6c: During the high-level hyperinsulinemic-euglycemic clamp (Experiment-3) the Olanzapine-treated animals show significantly less hepatic glycogen storage than the control group (p = 0.043; T-test). Vehicle-treated animals  =  white bars; Olanzapine-treated animals  =  black bars. Abs/mg  =  Absorption measured at 600 nm per mg wet tissue.

#### 2. Experiments 2 and 3: Hyperinsulinemic-euglycemic clamps

Both hyperinsulinemic-euglycemic clamp experiments (Experiments 2 and 3) started with a continuous [6,6-^2^H_2_]glucose infusion and an intragastric vehicle infusion at t = 0. Blood samples were collected at t = −5 min for background enrichment, and at t = 90, t = 95 and t = 100 min to determine enrichment at the equilibrium state.

Starting at t = 100, insulin (Actrapid 100 IU/ml; Novo Nordisk, Alphen aan de Rijn, The Netherlands) was administered in a primed (7.2 mU/kg/min for Experiment 2 and 21.6 mU/kg/min for Experiment 3 during 5 min) – continuous (3 mU/kg/min for Experiment 2 and 9 mU/kg/min for Experiment 3) intravenous infusion. A variable infusion of a 25% glucose solution containing 1% [6,6-^2^H_2_]glucose was used to maintain euglycemia, which was checked every 10 min by blood sampling. Thirty minutes after the start of the insulin infusion, the intragastric vehicle infusion was replaced by Olanzapine or vehicle (36 mg/kg/h during 5 min and 3 mg/kg/h till the end of the experiment). At the end of the experiment, five final blood samples were collected with a 10 min interval from t = 250 till t = 290 to determine isotope enrichment during the hyperinsulinemic-euglycemic state. At the end of the experiment, animals were sacrificed by a lethal intravenous injection of pentobarbital. Trunk blood was collected for plasma Olanzapine measurement at the end of the experiment. Samples of liver were snap frozen in liquid nitrogen and stored for glycogen measurements.

### Laboratory Methods/Analysis

Glucose concentrations were determined using a glucometer (Abbott). Blood samples were collected in tubes containing heparin on ice and centrifuged at +4°C. Plasma was stored at −20°C until further analysis.

Plasma insulin and corticosterone concentrations were measured using radioimmunoassay kits (Millipore, Billerica, USA and MP Biomedicals, Orangeburg, USA, respectively). Plasma [6,6-^2^H_2_]glucose enrichment was measured by gas chromatography-mass spectrometry (GCMS), and EGP was calculated by the methods of Steele [26].

Hepatic glycogen content was measured as described before with minor adaptations [27]. Briefly, 10–20 mg snap-frozen liver tissue was homogenized in 1 ml 5% trichloroacetic acid and incubated for 30 min at room temperature. After centrifugation for 10 min, glycogen was precipitated from the supernatant by adding 2 volumes of 95% ethanol and centrifugation for 30 min. The supernatant was discarded and the precipitate dissolved in a 1∶60 dilution of Lugol’s reagent in 25% (wt/vol) potassium chloride containing 30 mM hydrochloric acid. The glycogen content was determined spectrophotometrically at 600 nm and normalized against the tissue weight.

Plasma Olanzapine concentrations were measured by LC/MS/MS coupled to Quattro I Xe, Using a 7 points calibration curve from 0.2 to 200 ng/ml and 3 quality standards (1–10 and 100 ng/ml) (Laboratoires Fournier, Solvay Pharmaceuticals now Abbot, Daix, France).

### Statistical Analysis

Data are expressed as mean ± standard error of the mean (SEM). Statistical analysis was performed using SPSS version 17.0. A p<0.05 was considered statistically significant.

For Experiment 1, an ANOVA with repeated measures was performed to compare glucose levels, endogenous glucose production, corticosterone levels and insulin levels. Plasma Olanzapine levels were compared using a One-Way ANOVA. For non detectable samples, 0.2 ng/ml (the detection level) was used for the statistical analysis. For Experiments 2 and 3, endogenous glucose production and glucose uptake levels were compared using an ANOVA with repeated measures and a post-hoc analysis using One-Way ANOVA. Plasma corticosterone and insulin levels were compared using ANOVA with repeated measures. Finally, liver glycogen contents were compared using a T-test.

## Results

### Experiment 1: Acute Intragastric Administration of Olanzapine Induces Hyperglycemia, Unlike ICV Administration

Plasma glucose concentrations were measured before and during the infusion of Olanzapine (intragastric infusion in Figure 1a, ICV infusion in Figure 2a). Control and Olanzapine groups showed similar basal blood glucose levels before the IG infusion started (i.e., 5.77±0.37 mmol/L for the control group and 5.98±0.17 mmol/L for the Olanzapine treated group). Intragastric infusion of Olanzapine in rats resulted in a significant increase (p<0.001) in glycemia 60 min after the start of the infusion, as compared to the control rats. Maximal glycemia levels were reached 2 h after the start of Olanzapine (8.68±0.43 mmol/L), while the control rats did not show a significant difference (6.15±0.26 mmol/L) (Figure 1a). ANOVA showed significant effects of *Time* (p<0.001), *Group* (p = 0.001) and Group * Time (p<0.001). Rats receiving an ICV Olanzapine infusion showed no significant difference in glycemia as compared to the control rats. Basal blood glucose levels were 5.51±0.26 mmol/L and 5.66±0.13 mmol/L and maximally increased to 6.13±0.17 mmol/L and 6.23±0.17 mmol/L during the infusion for the control and Olanzapine groups, respectively (*Group*, p = 0.635; Figure 2a).

Endogenous glucose production (EGP) was measured by calculating the ratio of labeled and unlabeled glucose in the plasma (Figure 1b for intragastric infusion and Figure 2b for ICV infusion). At the start of the intragastric infusion of Olanzapine, basal EGP was comparable for both groups (53.76±2.14 µmol.kg^−1^.min^−1^ for control animals and 60.49±5.8 µmol.kg^−1^.min^−1^ for Olanzapine animals, p = 0.687). Infusion of Olanzapine did not show any effect on EGP (*Group*, p = 0.356; Figure 1b). Also during the ICV infusion of Olanzapine, no significant changes in EGP were found (basal EGP 63.99±1.17 µmol.kg^−1^.min^−1^ for controls and 65.83±3.16 µmol.kg^−1^.min^−1^ for Olanzapine animals and, at t = 240, experimental EGP 69.9±9.92 µmol.kg^−1^.min^−1^ for controls and 65.7±3.42 µmol.kg^−1^.min^−1^ for Olanzapine animals; *Group*, p = 0.805; Figure 2b).

Corticosterone levels showed a steady increase over time in both intragastrically-treated groups, with a significant increase in the Olanzapine-treated animals (*Group*, p = 0.039; Figure 1c). In the ICV-treated groups no significant effects of *Time* or *Group* were found for the plasma corticosterone concentrations. Neither the intragastric nor ICV Olanzapine infusion affected plasma insulin levels (Figures 1d and 2d) nor glucagon levels (data not shown).

### Plasma Levels of Olanzapine After Intragastric and ICV Administration of Olanzapine

Plasma Olanzapine levels were assessed at the end of Experiment 1. Plasma Olanzapine concentrations after intragastric infusion of Olanzapine were 285.9±59.6 ng/ml for treated group and non detectable (i.e., limit of detection 0.2 ng/ml) for controls (p<0.001; Figure 3). After ICV administration, plasma levels of Olanzapine were significantly lower than after the peripheral infusion (*Administration route*; p<0.001) and there was no significant difference between ICV controls (<0.2 ng/ml) and ICV Olanzapine animals (1.78±0.84 ng/ml; p = 0.2; Figure 3).

### Experiment 2: Acute Intragastric Administration of Olanzapine Induces Hepatic Insulin Resistance

During the hyperinsulinemic-euglycemic clamp (Experiment 2) we assessed hepatic insulin sensitivity by raising the circulating plasma insulin concentration ∼30% above the basal concentration of insulin (Figure 4d). Glucose levels were successfully clamped at an average of 6.21±0.06 mmol/L, and no significant differences were noticed between the 2 groups (Time * Group, p = 0.723 and *Group,* p = 0.113; Figure S1). The glucose infusion rate (GIR) needed to maintain euglycemia was significantly lower in the Olanzapine-treated animals compared to vehicle-treated animals (Time * Group, p = 0.001; Figure S2). Basal EGP was identical for both groups (53.56±4.89 µmol.kg^−1^.min^−1^ for controls and 55.4±2.87 µmol.kg^−1^.min^−1^ for Olanzapine-treated rats). The physiological effect of a modest increase in circulating plasma insulin is evidenced by the 35% decrease of EGP in the control group (p = 0.018). The inhibitory effect of insulin was clearly reduced by the Olanzapine infusion resulting in a non significant 14% decrease of EGP (p = 0.111; Figure 4a). Vehicle-treated animals showed a lower EGP at the end of the insulin clamp compared to Olanzapine-treated animals (p = 0.06).

Glucose disappearance showed a similar pattern (Figure 4b). Basal levels were identical for both groups (53.56±4.89 µmol.kg^−1^.min^−1^ for controls and 55.4±2.87 µmol.kg^−1^.min^−1^ for Olanzapine-treated rats), but the insulin-induced increase of glucose uptake was much more pronounced in the control group (+26.57 µmol.kg^−1^.min^−1^) than in Olanzapine-treated animals (+13.49 µmol.kg^−1^.min^−1^; *Time* * *Group*, p = 0.048; Figure 4b).

The results of the corticosterone data show that while in the control group, plasma corticosterone concentrations during the experiment were quite stable, the Olanzapine-treated group showed a significant increase of their circulating corticosterone level (18.61±5.70 ng/ml at baseline and 314.14±53.46 ng/ml at the end of the experiment; *Time* * *Group*, p = 0.028; Figure 4c).

### Experiment 3: Acute Intragastric Administration of Olanzapine Induces Extra-hepatic Insulin Resistance

In order to investigate the effect of Olanzapine on glucose disappearance in more detail, we performed an additional clamp study, in which circulating insulin concentrations were increased >4-fold (Figure 5d). Glucose levels were successfully clamped at an average of 5.74±0.051, and no significant differences were detected between the 2 groups (Time * Group, p = 0.628 and *Group,* p = 0.631; Figure S3). The GIR needed to maintain euglycemia was close to zero in the Olanzapine group and significantly lower in the Olanzapine-treated compared to vehicle-treated animals (Time * Group, p<0.001; Figure S4). Basal EGP of the 2 groups did not differ (57.07±9.01 µmol.kg^−1^.min^−1^ for the controls and 44.26±2.40 µmol.kg^−1^.min^−1^ for the Olanzapine-treated group, p = 0.22). Control animals showed a strong decrease of EGP due to high plasma insulin levels, i.e. almost 75% inhibition. Olanzapine-treated animals, however, showed a milder decrease, i.e., ∼50% (*Time* * *Group*, p = 0.018; Figure 5a).

The glucose disappearance results showed a comparable pattern: similar basal levels, a large insulin-stimulated increase, but a smaller increase for the Olanzapine group (*Group,* p = 0.005; Figure 5b). Corticosterone data were comparable to the “low” clamp group. The control group showed a slight increase of corticosterone during the experiment (21.29±4.99 ng/ml at basal level and 87.42±21.99 ng/ml at the end of the experiment), whereas the Olanzapine-treated animals showed a significant increase of their circulating corticosterone levels (36.19±12.35 ng/ml at baseline and 271.45±51.22 ng/ml at the end of the experiment, *Group,* p = 0.035; Figure 5c).

### Effect of Acute Olanzapine Treatment on Glycogen Storage

Relative hepatic glycogen content was assessed in all 3 experiments (Figure 6). Although mean hepatic glycogen levels appeared to be higher in Olanzapine-treated animals in Experiment 1 (Figure 6a), this increase did not reach statistical significance (p = 0.096). During the “low” hyperinsulinemic-euglycemic clamp (Experiment 2), the insulin infusion resulted in a physiologic increase in glycogen storage in the control group (as compared with the non-insulin treated animals in 6a). However, hepatic glycogen levels in Olanzapine-treated animals were lower than in the control group (p = 0.007) (Figure 6b). During the “high” hyperinsulinemic-euglycemic clamp (Experiment 3), hepatic glycogen content of Olanzapine-treated animals again were significantly lower than those of the control group (p = 0.043) (Figure 6c).

## Discussion

In this study, we compared the effects of peripheral and central administration of Olanzapine on glucose metabolism. Acute intragastric administration of Olanzapine clearly mimicked the adverse metabolic side-effects known from patients, as it induced both hyperglycemia and insulin resistance, both at hepatic and extra-hepatic levels. Acute ICV administration of Olanzapine did not result in any of these changes, indicating that the initiation of the metabolic side-effects of Olanzapine is mainly based on a peripheral mechanism. Moreover, our results show that the unfavorable effects of Olanzapine can occur independently of weight gain.

### Olanzapine Side-effects: Central vs Peripheral?

Recently Martins et al. (2010) compared the effects of intravenous (i.v.) and ICV infusion of Olanzapine on insulin sensitivity. While the results of their i.v. administration were comparable to our IG administration, the ICV results of that study are in clear contrast with the current study. Similar to the subcutaneous administration [28] and i.v. administration [24] of Olanzapine, our intragastric route of administration also resulted in hepatic and extra-hepatic insulin resistance. On the other hand, contrary to the lack of effect of ICV administration of Olanzapine in our study, Martins et al. (2010) also reported an induction of hepatic insulin resistance with ICV administration. However, there are several differences in the experimental design that might explain these contrasting results. Most importantly, the dose of Olanzapine used by Martins et al. (2010) was almost 10-fold higher than the dose used in this study (i.e., 330 µg/rat for Martins et al. (2010) and a total of 36 µg/rat in this study). Another study reporting the ICV administration of Olanzapine [25] showed a transient sedative effect after using a 50 µg/rat dose but none at 20 µg/rat. Our intermediate dose (36 µg/rat) indeed showed no sedative effects, but clearly part of the effects in Martins’ study might be linked to sedation [24]. Next, we implanted our ICV cannulas into the lateral ventricle instead of the third ventricle, which was targeted in the study of Martins et al. (2010). Several hypothalamic nuclei, involved in the control of energy metabolism, are located close to the borders of the third ventricle. Therefore, in the Martins’ study these hypothalamic nuclei probably have been exposed to much higher concentrations of Olanzapine than in our study [24]. Finally, our study was performed in male Wistar rats and Martins et al. (2010) used male Sprague Dawley rats. The measurements of the plasma Olanzapine concentrations that we performed in samples taken immediately at the end of the infusion showed a small, but detectable, amount of Olanzapine (1.8±0.8 ng/ml) after ICV administration, i.e. ∼0.5% of those in the IG Olanzapine group (∼280 ng/ml). In IG and ICV vehicle groups, plasma Olanzapine concentrations were below the detection level. Unfortunately, in the study of Martins et al. (2010) no plasma Olanzapine measurements were provided. However, since even with our low dose, Olanzapine passed from the ventricle to the plasma, higher doses of Olanzapine most likely will increase the level of Olanzapine in plasma even further and thus make it very difficult to distinguish between a central and a peripheral effect. In the light of the current data, it is plausible that a combination of the 3^rd^ ventricle injection site and a 10-fold higher dose would possibly result in a leakage of Olanzapine to the peripheral circulation. Therefore, the conclusion that metabolic side-effects of ICV Olanzapine are mediated by the hypothalamus may be erroneous.

### Peripheral Olanzapine Induces Hyperglycemia and Insulin Resistance

Intragastric Olanzapine infusion rapidly influenced glucose metabolism, as in basal conditions blood glucose concentrations were already increased by 1 mmol/L only 60 minutes after the start of the infusion. The clamp experiments (Experiments 2 and 3) showed a clear hepatic and extra-hepatic insulin resistance that likely contributes to the increased glycemia following Olanzapine infusion. Since in Experiment 1 hepatic glucose production was not significantly increased this would indicate a non-hepatic reduction in glucose uptake as the primary mechanism of Olanzapine to induce hyperglycemia. Corticosterone levels of Olanzapine-treated animals were elevated, but it is unlikely that this hormone causes the observed hyperglycemia as the increase was not sufficient to induce changes in hepatic glucose production. On the other hand, glucocorticoids are also involved in the regulation of insulin secretion [29], [30], and, although in our study plasma insulin levels remained unchanged during Olanzapine treatment, the lack of an increased insulin secretion could facilitate the hyperglycemic effect of Olanzapine. Moreover, it has also been shown that glucocorticoids like corticosterone can impair insulin signaling, primarily by decreasing total IRS1 protein expression and increasing Ser^307^ phosphorylation. IRS1 serine phosphorylation at this site had been reported to decrease the affinity of IRS1 for the insulin receptor and increase IRS1 degradation [31], which seems sufficient to account for the glucocorticoid-induced decrease of insulin-stimulated glucose uptake in skeletal muscle [29].

The clinical syndrome of glucocorticoid excess, Cushing’s syndrome, includes decreased insulin sensitivity, hyperglycemia and diabetes. Recently, it has been shown that selective glucocorticoid receptor (type 2) antagonists are able to prevent the weight gain in rats induced by chronic Olanzapine treatment [32]. Belanoff et al. (2011) suggest that, since the weight gain reduction is not mediated by a decrease in food intake, the selective glucocorticoid receptor (type 2) antagonist might act on metabolic processes rather than controlling appetite. However, the mechanisms underlying glucocorticoid antagonist mediated inhibition of weight gain have not been fully elucidated yet. Nevertheless, these data indicate the glucocorticoid receptor as an interesting target for preventing the metabolic side-effects of Olanzapine.

### Conclusion

In conclusion, we show that the primary side of action for the metabolic side-effects of Olanzapine are based on a peripheral mechanism, since ICV treatment did not result in any acute glucoregulatory changes. Nevertheless, it is still possible that subsequent to these primary events in the periphery an afferent signal is transmitted to the central nervous system [33] and central mechanisms are thus implicated in subsequent steps of the metabolic side-effects of Olanzapine.

## Supporting Information

Figure S1 **Plasma glucose levels during Experiment 2.** Plasma glucose levels of the control (open dots) and Olanzapine-treated animals (closed dots) did not significantly differ during the clamp experiment (ANOVA repeated measures; Time * Group, p = 0.723; *Group,* p = 0.113).
(TIF)

Figure S2 **Glucose infusion rate during Experiment 2.** The glucose infusion rate in the Olanzapine-treated animals (closed dots) is significantly lower than that in the control group (open dots) (ANOVA repeated measures; *Time,* p<0.001; Time * Group, p = 0.001; *Group,* p = 0.191).
(TIF)

Figure S3 **Plasma glucose levels during Experiment 3.** Plasma glucose levels of the control (open dots) and Olanzapine-treated animals (closed dots) did not significantly differ during the clamp experiment (ANOVA repeated measures; Time * Group, p = 0.628; *Group,* p = 0.631).
(TIF)

Figure S4 **Glucose infusion rate during Experiment 3.** The glucose infusion rate in the Olanzapine-treated animals (closed dots) is significantly lower than that in the control group (open dots) (ANOVA repeated measures; *Time,* p<0.001; Time * Group, p<0.001; *Group,* p = 0.245).
(TIF)
