# Supplementary figures and images for: Acute Peripheral but Not Central Administration of Olanzapine Induces Hyperglycemia Associated with Hepatic and Extra-Hepatic Insulin Resistance
Source: PLoS One. 2012 Aug 14;7(8):e43244. doi: 10.1371/journal.pone.0043244 (PMC3419184; doi:10.1371/journal.pone.0043244)

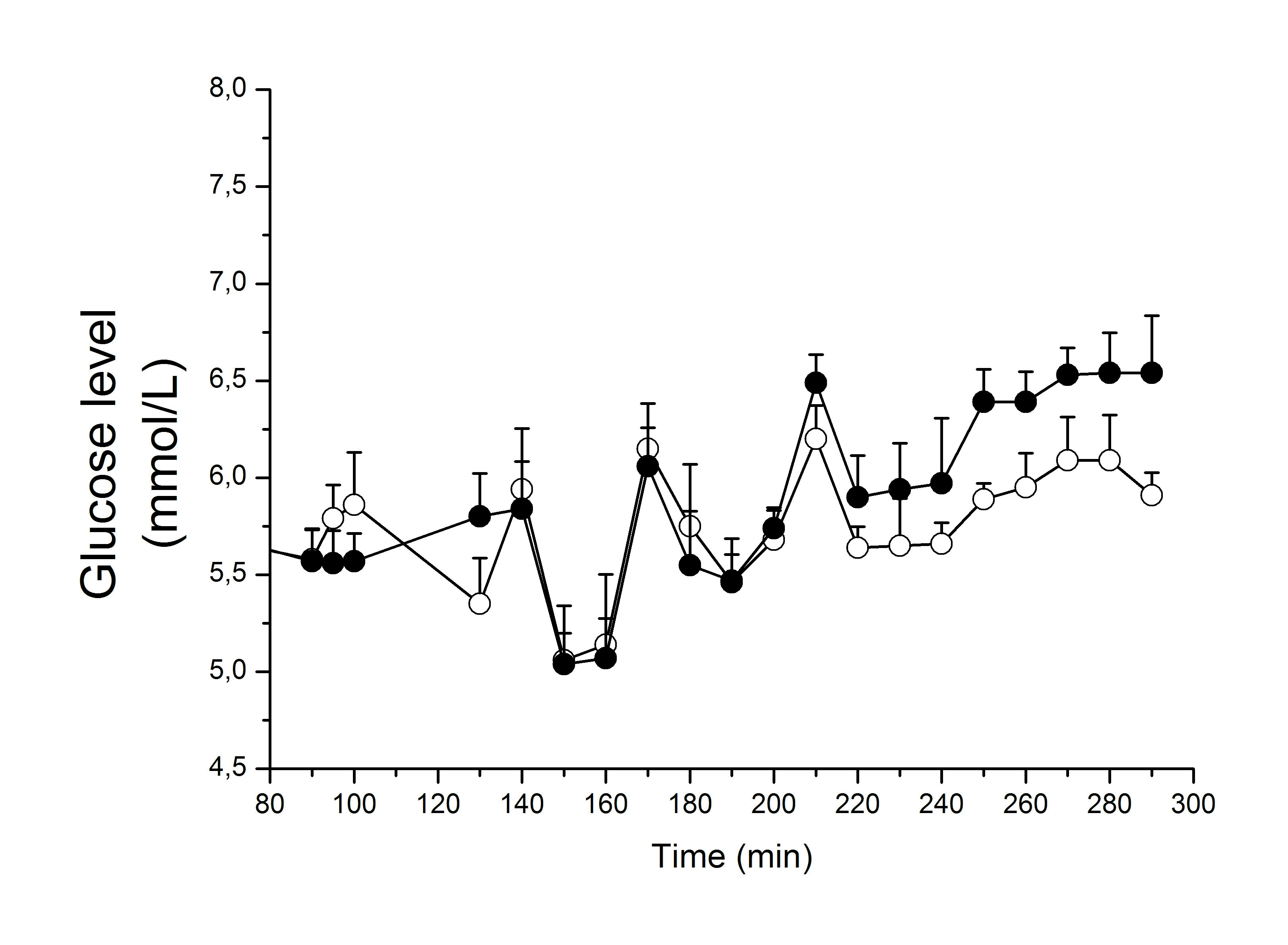

Supplement: Figure S1 — Plasma glucose levels during Experiment 2. Plasma glucose levels of the control (open dots) and Olanzapine-treated animals (closed dots) did not significantly differ during the clamp experiment (ANOVA repeated measures; Time * Group, p = 0.723; Group, p = 0.113). (TIF) [file pone.0043244.s001.tif]

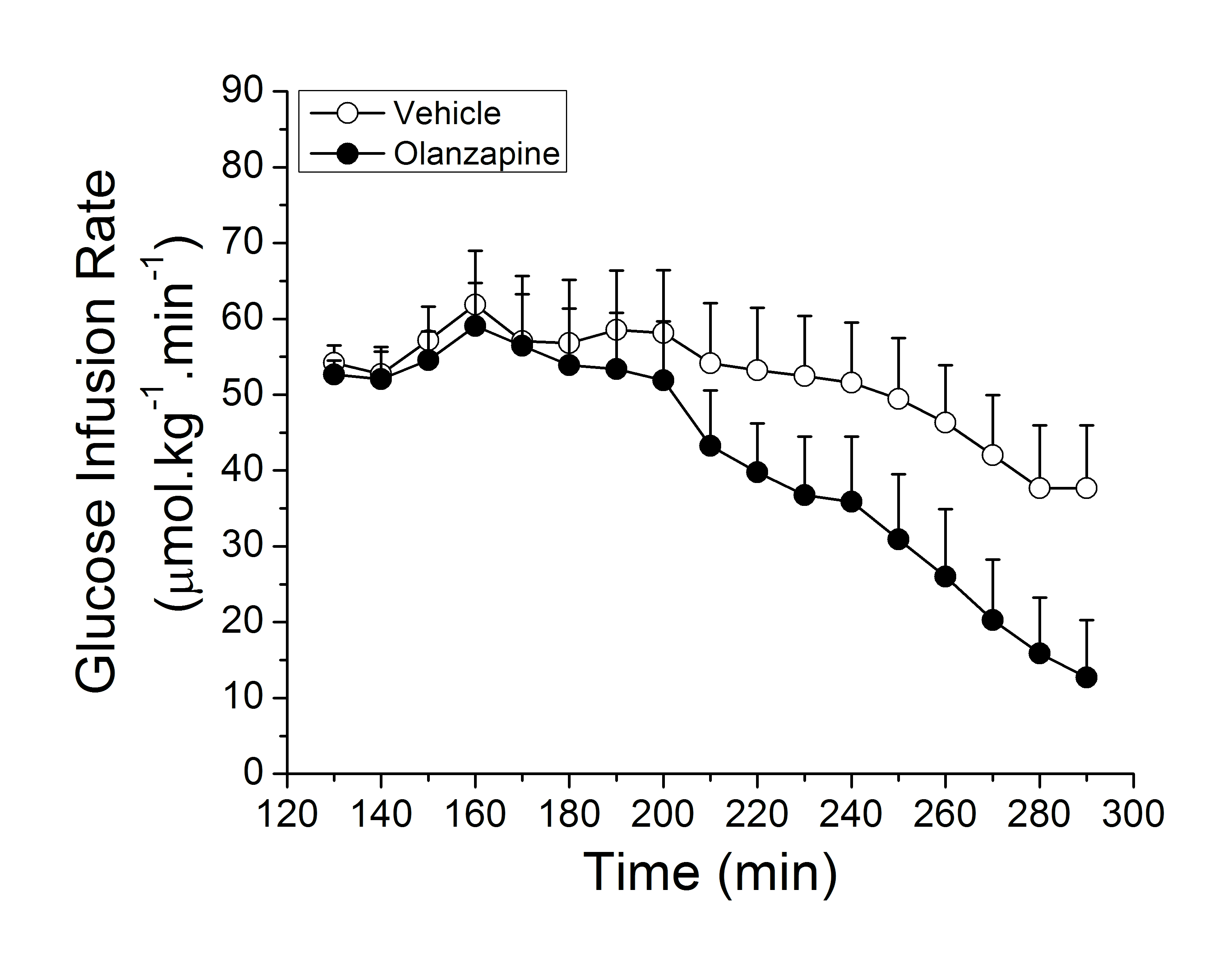

Supplement: Figure S2 — Glucose infusion rate during Experiment 2. The glucose infusion rate in the Olanzapine-treated animals (closed dots) is significantly lower than that in the control group (open dots) (ANOVA repeated measures; Time, p<0.001; Time * Group, p = 0.001; Group, p = 0.191). (TIF) [file pone.0043244.s002.tif]

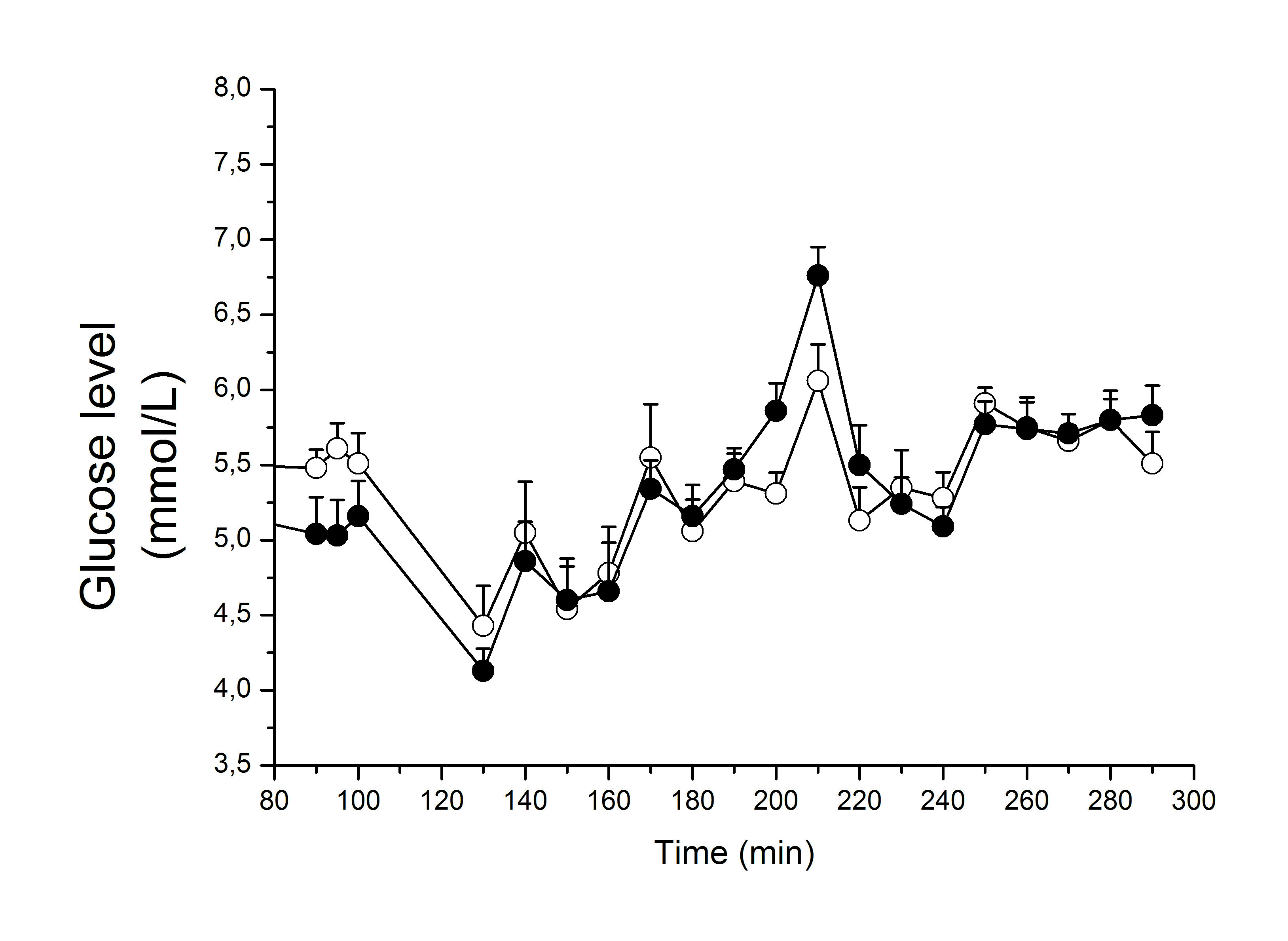

Supplement: Figure S3 — Plasma glucose levels during Experiment 3. Plasma glucose levels of the control (open dots) and Olanzapine-treated animals (closed dots) did not significantly differ during the clamp experiment (ANOVA repeated measures; Time * Group, p = 0.628; Group, p = 0.631). (TIF) [file pone.0043244.s003.tif]

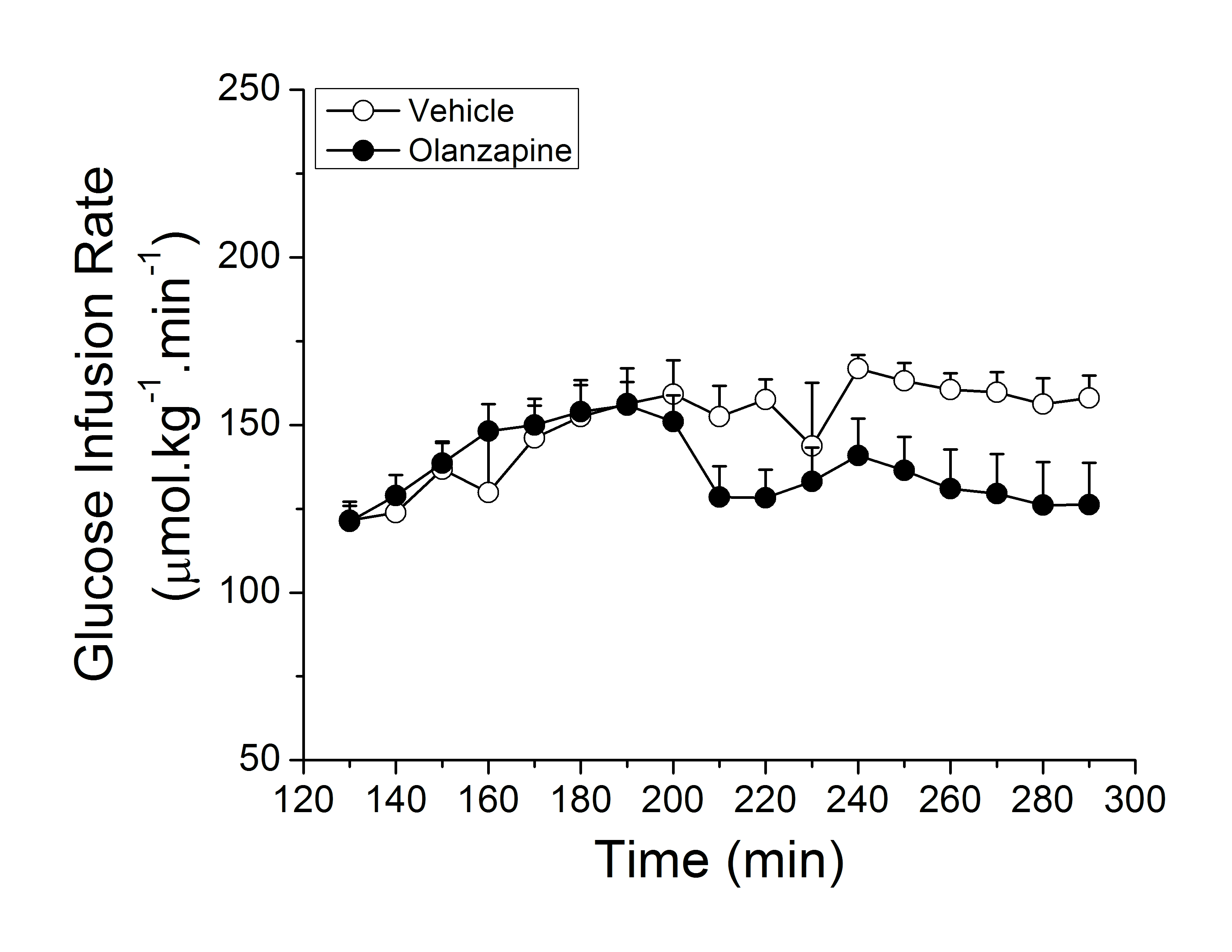

Supplement: Figure S4 — Glucose infusion rate during Experiment 3. The glucose infusion rate in the Olanzapine-treated animals (closed dots) is significantly lower than that in the control group (open dots) (ANOVA repeated measures; Time, p<0.001; Time * Group, p<0.001; Group, p = 0.245). (TIF) [file pone.0043244.s004.tif]
